# Psychometric evaluation of the Decision Support Tool for Functional Independence in community-dwelling older people

**DOI:** 10.1007/s12062-022-09361-x

**Published:** 2022-03-29

**Authors:** S. C. van Bijsterveld, J. A. Barten, E. A. L. M. Molenaar, N. Bleijenberg, N. J. de Wit, C. Veenhof

**Affiliations:** 1 Physical Therapy Sciences, Program in Clinical Health Sciences, University Medical Center Utrecht, Utrecht University, Utrecht, The Netherlands; 2 Department of Rehabilitation, Physical Therapy Science & Sports, University Medical Center Utrecht, Utrecht University, Utrecht, The Netherlands; 3 Research Group Innovation of Human Movement Care, Research Centre for Healthy and Sustainable Living, Utrecht University of Applied Sciences, Utrecht, The Netherlands; 4 Julius Center for Health Sciences and Primary Care, University Medical Center (UMC) Utrecht, Utrecht, The Netherlands; 5 Research Group Proactive Care in Older People, Research Centre for Healthy and Sustainable Living, Utrecht University of Applied Sciences, Utrecht, The Netherlands; 6 Center for Physical Therapy Research and Innovation in Primary Care, Julius Health Care Centers, Utrecht, The Netherlands

**Keywords:** Functional Independence, Psychometric Evaluation, Ageing, Community-Care, Older People, Prevention

## Abstract

**Background:**

The aging population is increasingly faced with daily life limitations, threatening their Functional Independence (FI). These limitations extend different life domains and require a broad range of community-care professionals to be addressed. The Decision Support Tool for Functional Independence (DST-FI) facilitates community-care professionals in providing uncontradictory recommendations regarding the maintenance of FI in community-dwelling older people. The current study aims to determine the validity and reliability of the DST-FI.

**Methods:**

Sixty community-care professionals completed a twofold assessment. To assess construct validity, participants were asked to assign predefined recommendations to fifty cases of older people to maintain their level of FI. Hypotheses were tested regarding the expected recommendations per case. Content validity was assessed by questions on relevance, comprehensiveness, and comprehensibility of the current set of recommendations. Twelve participants repeated the assessment after two weeks to enable both within- and between rater reliability properties, expressed by an Intraclass Correlation Coefficient.

**Results:**

Seven out of eight predefined hypotheses confirmed expectations, indicating high construct validity. As the recommendations were indicated ‘relevant’ and ‘complete’, content validity was high as well. Agreement between raters was poor to moderate while agreement within raters was moderate to excellent, resulting in moderate overall reliability.

**CONCLUSION:**

The DST-FI suggests high validity and moderate reliability properties when used in a population of community-dwelling older people. The tool could facilitate community-care professionals in their task to preserve FI in older people. Future research should focus on psychometric properties like feasibility, acceptability, and developing and piloting strategies for implementation in community-care.

## INTRODUCTION

With the population aged 65 and over growing faster than all other age groups, the world’s population is ageing. By the year 2050, one in four people living in North America and Europe is expected to be aged 65 or over (United Nations, 2019). With 26% of its inhabitants expected to be over 65 years of age by the year 2040, the country of The Netherlands is no exception to this international trend (RIVM, 2018). As age rises, so do impairments that hinder functioning in daily life. The Dutch National Institute for Public Health and the Environment estimated that in two decades, one in every three inhabitants will suffer from at least two chronic conditions (National Institute for Public Health & the Environment, 2018). The ageing population, the rising demand for care and the decreasing number of informal caregivers will challenge both Dutch society and its' healthcare system for at least the next twenty years (RIVM, 2018). To manage those challenges, a different approach has been implemented over the last years. This approach follows the renewed definition of health which aims for maintaining capabilities and independence, instead of mainly focusing on curing chronic diseases. (Huber et al., 2011). The combination of this more holistic view on health and the future healthcare challenges will have the focus of healthcare shift increasingly towards maintaining and facilitating older people as ‘functionally independent’ as long as possible (Government of the Netherlands, 2018; Huber et al., 2011; WHO, 2019).

**Fig. 1:**
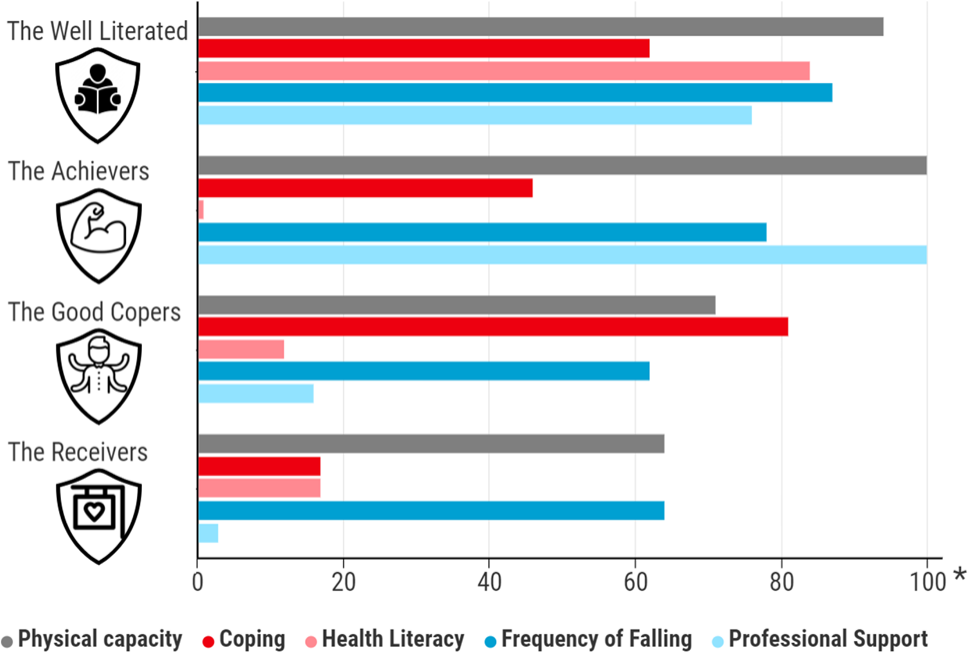
Characteristics of Profiles of Functional Independence. *Note*. * Figure indicating the percentage of people within each profile that score above cut-off points for every outcome. A low percentage on Frequency of Falling implies more people within that profile recently fell. A low percentage on Professional Support implies more people within that profile receive some form of care

The definition of functional independence has first been described by Kidd et al. in 1995 (Kidd et al., 1995). The current study uses the revised definition (scoping review by Molenaar et al. page 13) for Functional Independence (FI) in community-dwelling older people as “Functioning physically safe and independent from other persons, within one’s own context” (Molenaar et al., 2020a, b, c). The construct of FI comprises a complex interaction of both individual and social domains. In addition to physical capacity, empowerment, coping, health literacy and actual behavior act like building blocks that coherently with contextual factors add up to the construct of FI. Previous research has identified the most influential factors and has established four profiles representing distinct and gradually decreasing levels of FI (Molenaar EALM, Barten D, Veenhof C (2020b). The characteristics of each profile are shown in Fig. 1. For instance, the profile of the 'Well Literated' older people distinguishes itself by having above-average health literacy. Older people belonging to the 'Achievers' profile stand out for having relatively good physical capacity. The older people related to the 'Good Copers' profile stand out for well-developed coping strategies, despite their inferior physical capacity and health literacy. Finally, older people representing the 'Receivers' profile have poor scores on most of the domains of FI. Similarly, the ‘Receivers’ receive the greatest amount of professional support from healthcare professionals, more so than the other profiles. In short, as the characteristics of each profile of FI differ, so do the required community-care professionals needed for maintaining FI. Moreover, as FI reflects a multidimensional construct, professional support will often transcend the expertise of one particular professional. Although all community-care professionals may be considered capable of getting a first impression of an older adult’s level of FI, specific expertise is needed to support such an adult adequately. For example, a physical therapist or movement coach could be involved for physical capacity impairments. The social worker or welfare worker might be relevant for coping challenges. Professionals such as the general practitioner, practice-nurse, dietician or district nurse could support health literacy in a positive way. Lastly, among others, the occupational therapist and physical therapist might be of added value for people that often fall (Molenaar et al., 2020a). Ultimately, FI can rather be interpreted as an interdisciplinary responsibility calling for interdisciplinary collaboration. However, at this moment, interdisciplinary collaboration seems to be insufficient, as current community-care has older people sometimes receive different or even contradictory advice, depending on the healthcare professional they visit.

**Fig. 2:**
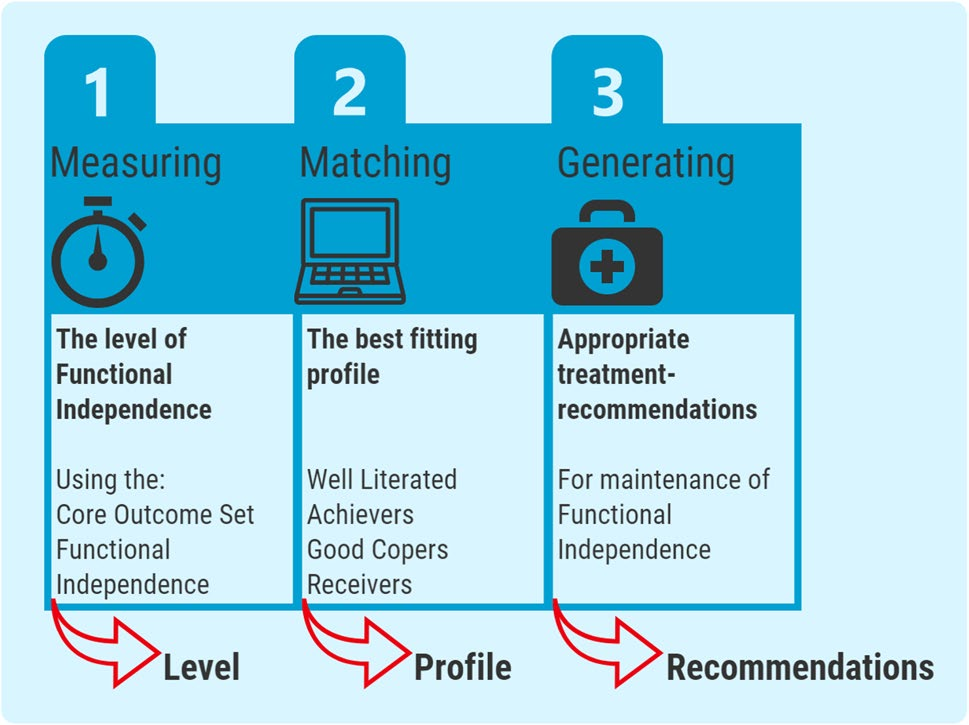
Mechanism of action of the Decision Support Tool for Functional Independence

Improved interdisciplinary collaboration regarding the maintenance of FI in community-dwelling older people requires a semantic unification among all the participants in the community-care playing field (Sangaleti et al., 2017). Improving the unity in language between healthcare professionals and narrowing gaps in knowledge on each specific healthcare professional could be accomplished by using a co-created, generally accepted tool that represents all relevant interdisciplinary views. In short, a tool is needed to provide healthcare professionals, especially community-care professionals, with recommendations regarding maintaining FI in community-dwelling older people. Prior research has shown that decision-making tools or decision-making trees can support transparency in clinical decisions, improve the delivery of personalized care and improve adherence to standards of care (Butera et al., 2019; Graham et al., 2018; Qin et al., 2016). Moreover, those tools have proven to be especially useful when multiple areas of expertise are included since it promotes shared decision-making (Davies et al., 2019; Manca et al., 2015). Given the evidence and need to support community-care professionals who aim to preserve FI in community-dwelling older people, the Decision Support Tool for Functional Independence (DST-FI) was recently developed in co-creation with potential users of this tool (e.g. community-care professionals) (Molenaar EALM, Barten D-J, Veenhof C (2020a). The DST-FI aims to assist community-care professionals by providing consistent recommendations regarding what older people need for preserving their level of FI. This tool covers all interdisciplinary aspects of FI and can be used by a broad range of community-care professionals. Figure 2 graphically outlines the mechanism of action of the DST-FI.

The content of the DST-FI has been established by way of representative interdisciplinary focus groups and, subsequently, a consensus meeting (Molenaar et al., 2020a). A more extended, external validation of these treatment-recommendations to be used by community-care professionals to preserve the level of FI in community-dwelling older people, is currently lacking. In order to use the DST-FI as both a valid and clinically relevant tool in a broad population of Dutch community-care professionals, psychometric evaluation of the DST over a wide spectrum of community-care professionals is required. Knowledge of validity and reliability measures is expected to promote the use and implementation of the DST-FI for the assessment of FI in community-dwelling older people (Lai, 2013).

Therefore, the objective of this study was to assess validity and reliability of the Decision Support Tool for Functional Independence (DST-FI) when used by community-care professionals in order to provide personalized recommendations regarding maintaining FI in community-dwelling older people, above 65 years of age.

## METHODS

### Study design

A cross-sectional descriptive psychometric evaluation study was conducted to determine the validity and reliability of the DST-FI (Part III) when used by community-care professionals in a population of community-dwelling older people. More specifically, this study focused on assessing content-validity, construct-validity, inter-professional reliability in several community-care professionals. Lastly, intra-professional reliability was determined particularly for physical therapists as they are the leading professionals in maintaining FI in community-dwelling older people.

This study followed, where applicable, the guidelines of the Consensus-based Standards for the selection of health Measurement Instruments (COSMIN-initiative) (Mokkink et al., 2019).

### Population

Participants were community-care professionals involved in attaining and sustaining the health of community-dwelling older people in the Netherlands. Since FI is an interdisciplinary construct, different types of health professionals were invited to ensure a broad psychometric evaluation of the DST-FI. Participants needed to either act as a general practitioner, practice-nurse, physical therapist, occupational therapist or as a district nurse. Exclusion criteria were; insufficiently digitally skilled, insufficient knowledge of the Dutch language or having no access to a laptop or pc with an internet connection.

The intended sample size of this psychometric study reflected the recommendations of the COSMIN initiative, thus aiming to analyze data of more than fifty community-care professionals to acquire adequate psychometric measures (Mokkink et al., 2019). As physical functioning is a key domain of FI, recruitment specifically focused on physical therapists (Molenaar et al., 2020c). Potential participants were approached by contacting professional-associations, community-care practices and individual professionals throughout the Netherlands. Recruitment took place via email, phone calls, and social media posts, in March and April of 2020. The originally planned on-site recruitment through presentations of the project leader was not possible due to social-distance regulations during the COVID-19 pandemic (National Institute for Public Health & the Environment, 2020). Therefore, online flyers were distributed instead.

### Explanatory – Decision Support Tool Functional Independence

The DST-FI comprises three subsequent parts. The first part concerns the measurement of older people’s level of FI by way of the Core Outcome Set Functional Independence (COSFI). The COSFI covers seven commonly used validated instruments and represents all separate domains of FI. The COSFI has shown to be able to distinguish between different levels of FI (Dockx et al., 2020). In addition to the COSFI, insight into the received level of professional caregiver support was acquired and a screening of history of falling was performed.

In the second part of the DST-FI, an older person will be matched to one of four profiles of FI, based on dichotomized outcomes of the COSFI. The profile which matches the most to an older person’s COSFI scores will be adopted. 

The third and final part of the DST-FI comprises generating appropriate treatment-recommendations based on the profile assigned in step two, which could then be used by community-care professionals. An example of such a generated recommendation contains the involvement of an occupational therapist.

### Study procedures

**Fig. 3:**
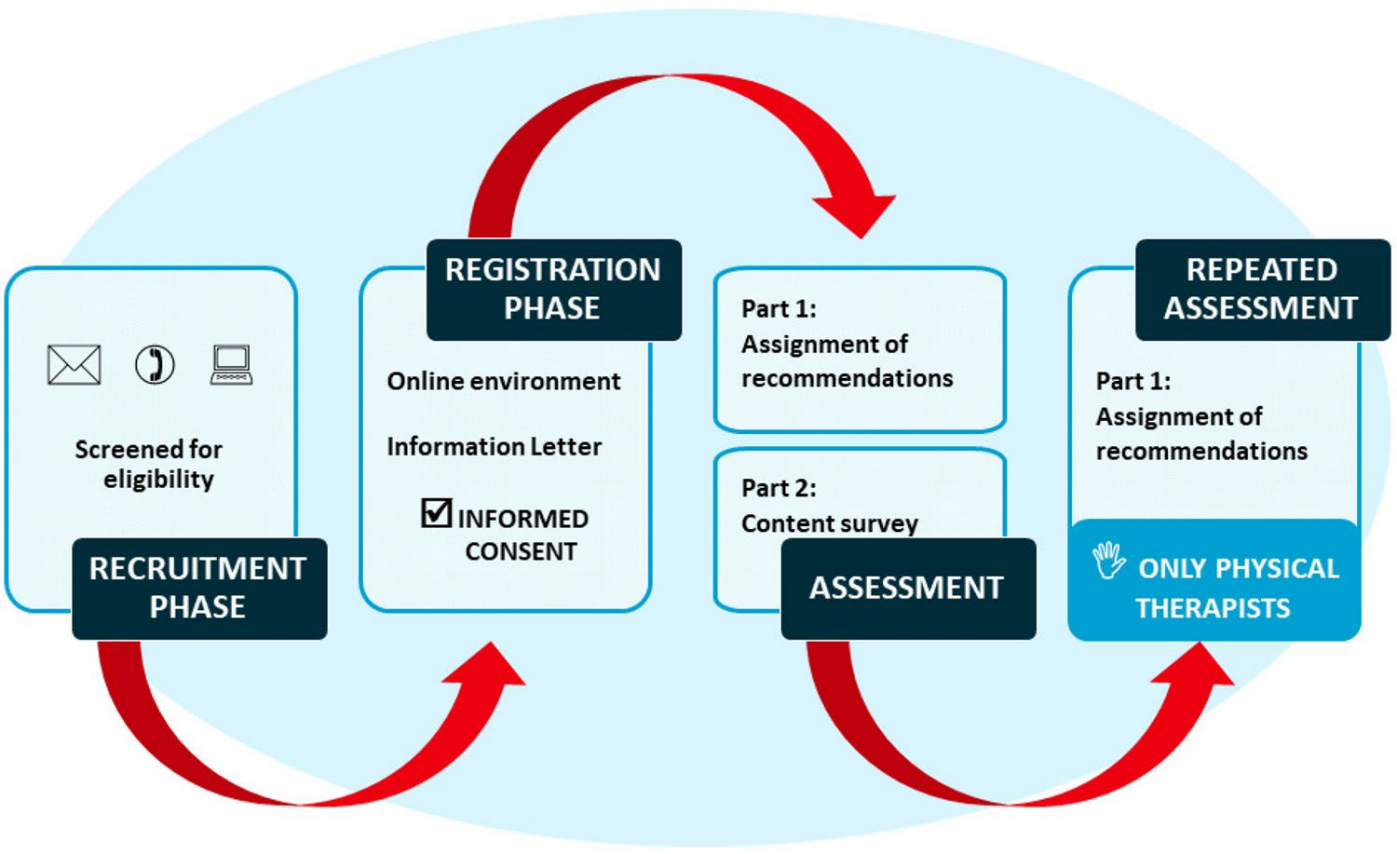
Flowchart of Study Procedures

The complete procedure of this study was graphically outlined in Fig. 3. All participants were asked to perform a one-time, online assessment consisting of two parts.

In the first part, participants were asked to recommend which community-care professionals should be involved in treating fifty unique cases of older people. These cases represented community-dwelling older people who have previously undergone COSFI-testing (Dockx et al., 2020). In the context of the current study, these cases of community-dwelling older people were anonymized.^1^ To ensure participants were able to form a picture of each older adult, every case of older adults comprised demographic data and information on the older people’s level of FI. More specifically, the following demographics were given: age (years), sex (male/ female), and living situation (alone/ with partner/ in a residential care center). Furthermore, information on the different domains of FI (physical capacity, health literacy, coping, frequency of falling and professional support) was given on a 3-point scale; either below average, average, or above average. See Appendix 1 for an example case of the online assessment. Based on this information, participants were asked to recommend a minimum of one and a maximum of four pre-defined community-care professionals that most suited the needs of each specific case. Participants selected up to four but did not need to put them into order. For example: when participants wanted only one professional to be involved, they needed to select ‘no further recommendations’ three times for analytical purposes. Possible community-care professionals to be recommended were: general practitioner, practice-nurse, district nurse, occupational therapist, physical therapist, welfare worker, movement coach, an interdisciplinary community-care team or a dietician.

In the second part of the assessment, participants completed questions on the relevance, comprehensiveness, and comprehensibility of the pre-defined set of community-care professionals (the recommendations) to be involved. For relevance, participants were asked to assign social roles to each professional that could be chosen during the first part of the assessment. Roles to choose from were: identify, initiate, execute, refer, and coordinate. Each professional could have multiple roles assigned. For comprehensiveness, participants were asked whether the current set of recommendations was complete. They answered on a five-point Likert scale (very incomplete, incomplete, mediocre, complete, very complete). Lastly, for comprehensibility of the response-set, participants were asked which potentially involved professionals they would like to be added to the set. Furthermore, participants had the chance to make comments on the procedure of assigning community-care professionals to cases based on FI deficiencies.

In addition to the primary assessment, for intra-rater reliability purposes, participants working as physical therapists were again asked to recommend which community-care professionals should be involved in maintaining older people’s FI. Instead of the complete set of fifty unique cases of older people, physical therapists completed the repeating assessment for the first 25 cases of the prior assessment. A minimum sample of 22 measurement units is required to determine an ICC of > 0.5 (Bujang, 2017). On top of that, a short questionnaire decreases the probability of missing data and thus was a pragmatic choice as well.

The online assessment was pilot-tested by community-care professionals and physical therapy students before the data collection took place. During this phase, the form was tested on clarity, interim saving, and time-to-complete (De Vet et al., 2011). The main findings of the pilot-testing were: unclear instructions on the assignment and inadequate description of the community-care professionals whose involvement could be recommended. Due to the COVID-19 restrictions, data-collection proceeded exclusively online.

### Outcomes

Outcomes of this study were four psychometric properties, priorly defined by the COSMIN group (Mokkink et al., 2010).

#### Construct validity

In the light of the DST-FI, *construct validity* refers to the degree to which treatment-recommendations generated by the DST-FI reflect the recommendations provided by the study participants. Assessing construct validity requires a-priori stated hypotheses about differences between groups to be tested. In this study, eight hypotheses were defined a-priori by the research team through a consensus meeting. These hypotheses were based on the characteristics of the four profiles (see Table 1) and included the expected direction of differences in recommended professionals to be involved per profile. Construct validity is considered ‘good’ when more than 50% of predefined hypotheses are confirmed (Mokkink et al., 2018).

**Table 1:** Hypotheses regarding the expected distribution of recommended professionals to maintain functional independence in older people

Expected (direction of) differences			Motivation	
1	It is hypothesized that	… the Achievers will receive more recommendations concerning the social worker than the Well Literated	based on	Their lower health literacy
2		…the Good Copers will receive more recommendations concerning an occupational therapist than the Well Literated		Their higher frequency of falling
3		… the Well Literated will receive more recommendations concerning the movement coach than the Achievers		Their slightly lower physical capacity
4		… the Receivers will receive more recommendations concerning the district nurse than the Achievers and the Well Literated		Their higher healthcare usage
5		… the Good Copers will receive more recommendations concerning a physical therapist than the Achievers		Their lower physical capacity
6		… the Receivers will receive more recommendations concerning a general practitioner than the Well Literated		Their higher healthcare usage and lower health literacy
7		… the Receivers will receive more recommendations concerning an occupational therapist than the Well Literated		Their higher frequency of falling
8		… the Receivers will receive more recommendations concerning the physical therapist than the Achievers		Their lower physical capacity

#### Content validity

*Content validity* covers the extent to which the current set of treatment-recommendations covers all domains of the FI-construct. The content validity of an instrument is made up of the degree of relevance, comprehensiveness, and comprehensibility (Terwee et al., 2018). However, the current study focused solely on the content of the set of professionals that could be involved. This study had participants complete a survey on the quality of the set of recommendations used in the DST-FI (i.e. the possible community-care professionals that could be chosen to be involved). Survey results were judged resulting in a descriptive rating of the content validity (regarding relevance, comprehensiveness, and comprehensibility of the set of recommendations). In summary, the aim was to make an objective description of whether participants agreed that the professionals incorporated in the DST adequately covered the treatment of older people with decreased FI.

#### Reliability

*Reliability* refers to the degree to which the recommended professionals differ between participants in this psychometric evaluation. The difference between participants is referred to as *interrater reliability* and the difference within participants over time is referred to as *intra-rater reliability* (Mokkink et al., 2010). The assessment of validity- and reliability measures of the DST-FI reflected the methodological guidelines of the COSMIN study design checklist (Mokkink et al., 2019). Assessing reliability measures required the results of individual participants to be compared. Briefly, if most participants independently from one another recommend more or less the same professionals to be involved, good reliability measures are to be expected (Mokkink et al., 2010). Assessment results of individual participants were compared per profile of FI. Therefore, recommended professionals to cases of the same profile were cumulated. For interrater reliability, the intraclass correlation coefficient (ICC) was used as a measure of similarity between participants (Koo & Li, 2016). Interrater reliability was determined 1) between raters with the same profession and 2) between all raters. Both were determined in the assumption that interrater agreement limited to one profession would be greater than the agreement between the entire group of raters since they had different areas of expertise. Thereafter, intra-rater reliability was determined as ICC. The ICC ranges from 0 to 1 and uses the following classification: values < 0.5 representing poor, 0.5–0.75 indicate moderate, 0.75–0.9 indicate good and values > 0.9 indicate excellent reliability (Koo & Li, 2016). As physical therapists are key professionals in maintaining FI in older people, these participants have been selected to determine intra-rater reliability.

### Statistical analyses

Although only entirely completed assessments were used for further analysis, descriptive statistics were used to get insight into the number of participants that either attempted or completed the analysis. These statistics were then stratified per profession. Partially completed assessments could not be used since analytical methods required an assumption to be met stating that all participants needed to have an equal number of ratings performed for every case rated (Koo & Li, 2016; Landers, 2015). Imputation of incomplete assessments was not suitable because all cases of older people were unique and their input contained multiple-response sets of answers (Jakobsen et al., 2017). To get insight into which community-care professionals were recommended to get involved for each case, thus for each profile, multiple-response sets were defined and frequency tables were generated (Miller et al., 2002a, 2002b).

Analysis for *construct validity* started with getting insight into whether or not significant deviations occurred in the distribution of recommendations between different profiles of FI. Pearson Chi-Square tests were used to test the null hypotheses of no relationship between profiles and assigned recommendations (Beasley & Schumacher, 1995). To correct for multiple testing and get insight into the between-group differences, adjusted standardized residuals were acquired and used for post-hoc cellwise residual analysis (García-Pérez & Núñez-Antón, 2003). The Bonferroni-corrected significance values combined with multiple-response frequency tables provided the information needed to either confirm or reject the a-priori stated hypotheses. A significance value of α = 0.05 was used.

Analysis for *content validity* started with describing answers to the survey’s questions regarding relevance, comprehensiveness, and comprehensibility. Answers to the two open questions were recited using straightforward codebook analysis. The questions on completeness of the current set of possible professionals to be involved and on social roles to be assigned to each professional were analyzed using frequency cross-tables. Lastly, the output of all four questions was used for the descriptive rating of the content validity of the DST-FI (Terwee et al., 2018).

Analysis for *reliability* measures required the frequencies of individual community-care professionals recommended to each profile to be compared among all participants. The similarity in recommended professionals between participants was determined for all participants and per profession. For interrater reliability, a two-way random-effects model was used and a consistency ICC was calculated (Koo & Li, 2016). For intra-rater reliability, a two-way mixed-effects model was used and an absolute agreement ICC was calculated, resulting in separate ICC values for each participant. (Koo & Li, 2016). All data were analyzed using Statistical Package for the Social Sciences (SPSS version 26.0, IBM Inc., Armonk NY, USA).

### Ethics

An online informed consent was collected by a checked-box prior to the online assessment. All personal data entered by participants was handled according to the rules of the General Data Protection Regulation (GDPR) (Van Alsenoy, 2019).

## RESULTS

### Population

Recruitment resulted in 88 professionals participating in the online assessment. Due to not-at-random missing data, assessments of 60 participants were included for analyses. This group consisted of seven general practitioners, ten occupational therapists, seven practice-nurses, 23 physical therapists, and thirteen district nurses. All five professions had a completion rate of 71% to 87% except for the district nurses which had a completion rate of 45%. As intended, physical therapists were the best-represented group of participants with a total of 23 completed assessments. Moreover, twelve physical therapists repeated the (shortened) assessment two weeks after completion of the primary assessment to assess intra-rater reliability.

### Construct validity

Table 2 shows absolute and relative frequencies of recommended professionals to be involved and the profiles to whom the assessed cases belonged. When a-priori stated hypotheses (see methods section) regarding the expected distribution of recommended professionals to profiles were compared with the findings in table 2, seven of the eight hypotheses were confirming expectations. Only hypothesis no. 3 concerning the distribution of movement coach recommendations was rejected, as shown in Table 3. Bonferroni corrected Pearson Chi-square tests showed no significant differences regarding the recommendation of movement coach involvement among the four profiles.

**Table 2:** Comparison of proportions of assigned recommendations to cases in each profile of functional independence.

Recommendations	Profiles							
	The Well Literated *11 cases*		The Achievers *10 cases*		The Good Copers *19 cases*		The Receivers *10 cases*	
	N	Percent	N	Percent	N	Percent	N	Percent
No additional recommendations	1288	49*	877	37*	749	16*	162	7*
General practitioner	216	8*	216	9*	503	11	377	16*
Practice-nurse	322	12	427	18*	612	13	328	14
Movement coach	123	5	123	5	188	4	103	4
Welfare worker	84	3	150	6*	126	3*	89	4
Social worker	131	5*	197	8*	279	6	171	7
Dietician	13	1*	55	2*	84	2	31	1
District nurse	200	8*	71	3*	858	19*	478	20*
Occupational therapist	111	4*	156	7*	475	10*	252	11*
Physical therapist	152	6*	128	5*	686	15*	409	17*
total	2640	100	2400	100	4560	100	2400	100

^N = absolute number of recommendations. Totals differ because cases are not evenly distributed over the four profiles. An asterisk implies a profile−recommendation relationship that deviates significantly from 'no difference'. Rounded to whole percentages.^

**Table 3:** Overview of tested hypotheses regarding recommendations of professionals to cases of older people

	Hypothesis	Confirmed
	It is hypothesized that…	
1	the Achievers will receive more recommendations concerning the social worker than the Well Literated	Yes
2	the Good Copers will receive more recommendations concerning an occupational therapist than the Well Literated	Yes
3	the Well Literated will receive more recommendations concerning the movement coach than the Achievers	No
4	the Receivers will receive more recommendations concerning the district nurse than the Achievers and the Well Literated	Yes
5	the Good Copers will receive more recommendations concerning a physical therapist than the Achievers	Yes
6	the Receivers will receive more recommendations concerning a general practitioner than the Well Literated	Yes
7	the Receivers will receive more recommendations concerning an occupational therapist than the Well Literated	Yes
8	the Receivers will receive more recommendations concerning the physical therapist than the Achievers	Yes

### Content validity

**Fig. 4:**
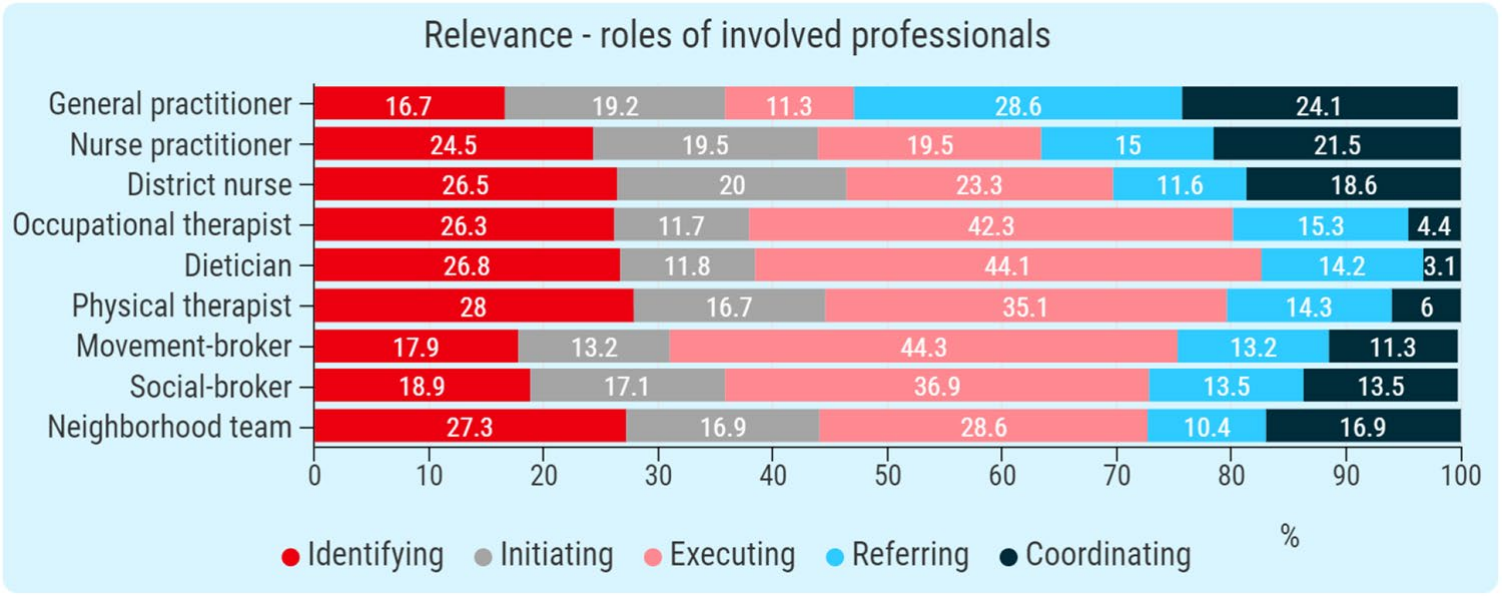
Content Validity of the set of involved professionals to maintain functional independence in older people—Relevance

Results of the survey on the quality of the response-set are shown in Fig. 4 and Table 4. In terms of relevance, all professionals were assigned multiple roles by participants. The general practitioner was mostly seen as coordinator and referrer. As shown in Fig. 4, multiple professionals were seen as identifiers, initiators or as executors. For comprehensiveness, a total of 51 out of 60 participants reported that the current set of recommendations is either complete or very complete. Lastly, for comprehensibility, several suggestions for additional professionals to be recommended and case-related information to be included were given, as shown in Table 4. For example, one participant has advised to make cognitive function part of the DST-FI and four participants have advised to include the psychologist as a recommendable professional.

**Table 4:** Content Validity of the set of involved professionals to maintain functional independence in older people—Comprehensiveness and Comprehensibility

Table 4a			Table 4b	
Comprehensiveness – completeness of response-set			Comprehensibility – additions and comments	
	Frequency	Percent	Suggestions for additional recommendations	
Very incomplete	0	0		Pharmacist (n = 1)
Incomplete	2	3.3		Case-manager dementia (n = 4)
Mediocre	7	11.7		Psychologist (n = 3)
Complete	42	70		Social worker (n = 2)
Very complete	9	15		Exercise therapist (n = 1)
Total	60	100	Suggestions for additional case-related information	Information on comorbidities (n = 2), body-composition (n = 2) and cognitive function (n = 1)

### Reliability

Reliability measures are shown in Table 5. Interrater reliability coefficients were *poor* to *moderate* and the mean intra-rater reliability coefficient was *good*. All findings were statistically significant. Although the mean intra-rater coefficient was classified as *good*, individual values ranged from *moderate* to *excellent*.

**Table 5:** Reliability measures – intra- and interrater

*Interrater Reliability*										
		Intraclass Correlation Coefficient		95% Confidence Interval						Agreement Classification
				Lower Bound	Upper Bound		F Test		P-value	
Overall Agreement (60 raters)		0.520		0.418	0.643		66.025		< 0.000*	Moderate
General practitioners (7 raters)		0.602		0.480	0.727		11.604		< 0.000*	Moderate
District nurses (13 raters)		0.523		0.409	0.653		15.246		< 0.000*	Moderate
Practice-nurses (7 raters)		0.656		0.540	0.769		14.347		< 0.000*	Moderate
Occupational therapists (10 raters)		0.595		0.480	0.717		15.687		< 0.000*	Moderate
Physical therapists (23 raters)		0.491		0.384	0.620		23.152		< 0.000*	Poor
*Intra-rater Reliability*										
Intraclass correlation coefficients (in physical therapists)	N	Minimum	Maximum			Mean		Standard Deviation		Agreement Classification
	12	0.637	0.963			0.839		0.104		Moderate to Excellent

^N = absolute number of repeated assessments by physical therapists. An asterisk implies a significant finding^.

## DISCUSSION

This study aimed to assess the validity and reliability of the DST-FI (Part III) when used by community-care professionals in a population of community-dwelling older people. Overall, the DST-FI has high validity measures. Regarding construct validity, seven out of eight hypotheses stating expected differences in the distribution of recommended professionals were accepted. For content validity, the current set of recommendations was rated as relevant and complete. Lastly, reliability measures showed to be moderate. However, it has to be mentioned that profession-specific interrater and intra-rater sample sizes did not meet COSMIN standards required for obtaining adequate measures.

An unexpected finding concerned the construct validity of the DST-FI. As opposed to our expectation stated in hypothesis no. 3, the Achievers (physically strong, older people) were more often associated with a movement coach than people represented by the Well Literated profile (high health-literacy level). Participants might have selected the movement coach to be involved in some cases from a preventive point of view. This corresponds to what is known about the role and context of movement coaches in The Netherlands, which states that the movement coach could help people maintain their level of physical activities (Leenaars, Smit, et al., 2018; Leenaars, van der Velden-Bollemaat, et al., 2018). Unfortunately, movement coaches were not participating in the current study for clarification of this issue. Furthermore, these results may suggest that many older people do not yet need community-care professional involvement and can maintain or improve their level of FI themselves. Moreover, a regularly scheduled FI-check might help a considerable group of older people to prevent future healthcare costs.

Another interesting finding concerned the interrater reliability measures. Most are classified as moderate and are therewith in line with previous, comparable psychometric evaluation studies (Butera et al., 2019; Qin et al., 2016). The interrater reliability in physical therapists however, as best represented group of participants and key players in maintaining FI in older people, appeared to be classified as poor. Unfortunately, it is difficult to explain these findings as we collected demographic data of the participants to a limited extent. In general, moderate scores on interrater reliability measures of the current study can be explained by several reasons. First and foremost, participants probably had different levels of experience and different areas of expertise. In addition, views on the added value of other, sometimes unknown professions might have differed among participants (Kozlowski et al., 2017). Moreover, the exposed differences in views between different professionals may reflect everyday clinical practice and its barriers (Doekhie et al., 2017; O’Reilly et al., 2017). Differences in views between professionals do not disqualify the use of the DST-FI because the tool as a whole seems to reflect the community’s view very well. Finally, the DST-FI will not replace the expertise of community-care professionals but facilitate their decision-making process, thus will always be used with a specific older adult in mind.

Regarding the content of the DST-FI, the pre-defined set of professionals to be involved was mostly rated as complete by the participating community-care professionals. Although participants suggested adding other professions to the set, current professions were all rated as relevant professions to treat older people with a (risk of) decreased level of FI. Furthermore, the current study shows each profile requires various professionals to get involved, therewith providing evidence for the interdisciplinary character of the construct of FI. This resembles previous studies stressing the importance of a multidisciplinary approach in the care for community-dwelling older people (Goodman et al., 2011; Tanaka, 2003; Trivedi et al., 2013).

### Strengths and Limitations

A strength of this study was the inclusion of a very diverse group of community-care professionals ensuring a wide clinical view on how to facilitate community-dwelling older people in maintaining their FI. Spread over five professions, sixty participants have completed the assessment which, according to COSMIN is an adequate representation. The proportion of physical therapists was quite higher than the other professionals. Although this may have influenced the outcome of the assignments, it does reflect the situation in clinical practice. Physical therapists are key players in maintaining FI in community-dwelling older people. For that reason, we did not account for the overrepresentation of physical therapists in our sample of community-care professionals. Furthermore, it should be mentioned that the completion rate was quite lower in the group of district nurses compared to the other groups of participants. This could be a consequence of the timing of the data-collection: COVID-19 had just made its appearance, which increased the workload of specifically district-nurses largely. Another strength comprised the applied procedure of psychometric evaluation. Psychometric evaluation of the DST-FI applied a rigorous procedure of testing eight priorly stated hypotheses that contained the expected direction of the results when comparing the different profiles of FI.

This study had some limitations that need to be considered. First and foremost, the hypotheses on expected differences in recommendations assigned to cases of the four profiles of FI only included the direction of expected differences between these profiles, whereas COSMIN calls for hypotheses including both a direction and a magnitude. Despite that we tried to argue specific magnitudes using existing data on characteristics per profile of FI, determining the magnitude of expected differences failed. Although cases within the same profile were similar in their shortcomings, they were not identical. Therefore, specific statements on the magnitude of inter-profile differences in recommendations assigned were nearly impossible to sufficiently substantiate. Further, the limited personal- and contextual information shown in the case description during the online assessment may have been a limitation in this study. By way of illustration, participants were able to recommend a dietician to get involved although they had no information on comorbidities, bodyweight or body-mass-index (BMI) of a specific case. Similarly, information on cognitive functioning was not given, despite cognitive function is known to have impact on an older adult’s level of FI (Dodge et al., 2006). Related to this, mental health professionals, such as a psychologist or case-manager dementia, may be added to the current set of recommendable professionals in an updated version of the DST-FI. Furthermore, the fact that no demographics of participating professionals were collected other than their profession, limits the ability to make statements on differences in their views. In hindsight, factors as age, sex, years of practice and workplace (rural or urban) might have explained some of the differences found, such as the poor interrater reliability measures in physical therapists. Data about these demographics could also have provided insight into the generalizability of the study findings.

### Recommendations

In this study, the DST-FI seems to be an appropriate instrument to adequately generate broadly-based recommendations regarding the involvement of community-care professionals to maintain FI in community-dwelling older people with a (risk of) decreased level of FI. However, several recommendations should be considered before starting to use the DST-FI in clinical practice.

First, although a moderate reliability trend held true for reliability in individual professions, sample sizes were too small to obtain adequate results. Therefore future studies on specific professionals are needed to confirm the findings of the current study.

Secondly, in response to participants that advised to incorporate more information and additional community-care professionals, the current scope of health domains of the DST-FI could be enriched. For instance, additional information that provides insight into body composition could aid the DST-FI in adequately recommending a dietician (Cruz-Jentoft et al., 2010; Morley, 2016). Similarly, if information on cognitive function would be given, this could assist the DST-FI in recommending a psychologist or case-manager dementia when required to sustain FI (Corvol et al., 2017; Lee & Waite, 2018; MacNeill & Lichtenberg, 1997). On the other hand, the community-care professional using the DST-FI will usually have more information about their client than just the recommendations generated by the DST-FI.

Thirdly, to ensure optimal applicability of the DST-FI, future research could focus on investigating the most feasible mode of use. Perhaps a digitally approachable form with a user-friendly graphical user interface will ease the use in clinical practice and enlarge opportunities for updating the DST-FI in the future (Henshall et al., 2019). Aside from feasibility, acceptability, and small-scale pilot testing should be considered. Moreover, the subsequent implementation process should involve several community-care professionals. Similar to the stages of development and validation, having a broad group of professions and older people themselves will benefit implementation in the care for community-dwelling older people (Luig et al., 2018).

Fourthly, although the current study provides considerable evidence for DST-FI usage in community-care, all participants were community-care professionals already involved in attaining and sustaining the health of community-dwelling older people. Therefore, further research should focus on assessing feasibility among a more diverse, group of community-care professionals who are less frequently involved in the care for older people. Ultimately, if the DST-FI proves to be suitable for professionals with varying experience working with community-dwelling older people, the DST will be of great value for the growing aging population.

## Conclusion

The DST-FI is suggested to be a highly valid and moderately reliable tool to generate treatment- recommendations regarding the maintenance of FI in community-dwelling older people. Future research is recommended on confirming the current result in larger samples of community-care professionals. To optimize its use in clinical practice, future research may be focused on widening the scope of included health-domains, assessing feasibility in less experienced community-care professionals and developing a user-friendly mode of use.

## Data Availability

Available upon request.

## Footnotes

1 The study described by Dockx et al. has obtained written informed consent from participants to join in the overarching research project. Ethics approval was performed by the ethical committee of the Utrecht University of Applied Sciences (Ref nr. 85–000-2019).

## Code availability

Available upon request.
